# Biochemical Characterization of Recombinant Thermostable *Cohnella* sp. A01 β-Glucanase

**DOI:** 10.29252/ibj.22.5.345

**Published:** 2018-09

**Authors:** Meysam Rezaie, Saeed Aminzadeh, Farid Heidari, Masoud Mashhadi Akbar Boojar, Ali Asghar Karkhane

**Affiliations:** 1 National Institute for Genetic Engineering and Biotechnology (NIGEB), Institute of Industrial and Environmental Biotechnology, Bioprocess Engineering Research Group, Shahrak-E-Pajoohesh km 15, Tehran-Karaj Highway, P. O. Box: 14965/161, Tehran, Iran; 2 Faculty of Biological Sciences, Kharazmi University, Tehran, Iran; 3 National Institute for Genetic Engineering and Biotechnology (NIGEB), Institute of Agricultural Biotechnology, Animal Biotechnology Department, Shahrak-E-Pajoohesh km 15, Tehran-Karaj Highway, P. O. Box: 14965/161, Tehran, Iran

**Keywords:** β-glucanase, Laminarin, Pustulan

## Abstract

**Background:**

Typically, non-cellulytic glucanase, including fungi and yeast cell wall hydrolyzing enzymes, are released by some symbiotic fungi and plants during the mycoparasitic fungi attack on plants. These enzymes are known as the defense mechanisms of plants. This study intends to investigate the biochemical properties of β-1,6-glucanase (bg16M) from native thermophilic bacteria, *Cohnella* A01.

**Methods:**

*bg16M* gene was cloned and expressed in *E. coli* BL21 (DE3). The enzyme was purified utilizing Ni-NTA nikcle sepharose column. Pustulan and laminarin were selected as substrates in enzyme assay. The purified bg16M enzyme was treated with different pH, temperature, metal ions, and detergents.

**Results:**

The expressed protein, including 639 amino acids, showed a high similarity with the hydrolytic glycosylated family 30. The molecular weight of enzyme was 64 kDa, and purification yield was 46%. The bg16M demonstrated activity as 4.83 U/ml on laminarin and 2.88 U/ml on pustulan. The optimum pH and temperature of the enzyme were 8 and 50 °C, respectively. The enzyme had an appropriate stability at high temperatures and in the pH range of 7 to 9, showing acceptable stability, while it did not lose enzymatic activity completely at acidic or basic pH. None of the studied metal ions and chemical compounds was the activator of bg16M, and urea, SDS, and copper acted as enzyme inhibitors.

**Conclusion:**

Biochemical characterization of this enzyme revealed that bg16M can be applied in beverage industries and medical sectors because of its high activity, as well as thermal and alkaline stability.

## INTRODUCTION

β-glucans are highly abundant in natural poly-saccharides and are found not only in plants but also mainly in some micro-organisms and a number of vertebrates. β-1,3-glucan and β-1,6-glucan are less common and are excreted by many fungi as a component of their cell wall and extracellular metabolites[1,2]. Some of the fungal β-D-glucans act as immunomodulators. Enzymatic modification of polysaccharides has introduced new applications for these modified polysaccharides in pharmaceuticals and foods industries. Gluco-oligosaccharides have health benefits that influence the immune system[3].

β-1,6-glucanase (bg16M, EC 3.2.1.75) is a non-cellulytic enzyme that hydrolyzes β-1,6-glycosidic bonds in different glucans. Based on amino acid sequences, the eukaryotic and prokaryotic bg16Ms are classified into glycosyl hydrolases 5 (GH5)[4] and glycosyl hydrolases 30 (GH30) families[5,6]. Enzymes of GH30 family hydrolyze β-glycoside bonds via acid-base mechanism, which was first proposed by Koshland[7]. This mechanism needs at least two amino acid residues in the active site of the enzyme, such that one acts as acid or base, and the other acts as a nucleophile[8]. These enzymes are categorized into glycosyl hydrolase A family, based on their three-dimensional structure, which is the triosephosphate isomerase barrel structure, and its active site is composed of two glutamic acid residues. Substrate of this enzyme is found in the cell wall structure of filamentous fungi[9] and yeasts[10], as well as in some fungi and lichens as secretory or storage polysaccharides such as pustulan (β-1,6-glucan)[11] and laminarin (β-1,3-1,6-glucan)[12,13].

In fungi, similar to other secretary hydrolase enzymes, bg16M helps to feed the fungus via the hydrolysis of glucans polymers in surrounding environment as well as participating in autolysis of fungus[14]. In general, bg16M has a great potential for medical applications, product management, and improvement of food quality, and it is also a suitable means for detecting cell wall components of fungi and yeasts[15].

Microorganisms such as *Aspergillus*, *Bacillus*, and *Trichoderma* are the most important sources of β-glucanase[13], but to date, based on our knowledge, only one active bacterial bg16M enzyme has been studied[16].

Among the enzyme-producing microorganisms, the thermophilic species are more considered in various industries because of their highly stable enzymes. In addition, catalysis of reactions at high temperatures decreases the risk of contamination and increases the solubility of organic compounds[17,18].

In the present study, heterologous expression and biochemical characterization of a novel thermostable bg16M from native thermophilic bacteria, *Cohnella sp*. A01, were carried out. However, in previous investigations, *Cohnella sp*. A01 lipase was studied[19], and the expression of a lipase gene from this bacterium was reported[20].

## MATERIALS AND METHODS

DNA extraction kits were purchased from Bioneer Company (Seoul, Korea). The restriction enzymes, *Nde*I, *Not*I, and plasmid pTZ57R/T were prepared from Fermentas (Glen Burnie, MD, USA). Plasmid extraction kit was procured from Roche Company (Switzerland). Bacterial strains including *E. coli* DH5α and *E. coli* BL21 (DE3), the expression vector pET-26b (+), and Ni-NTA resin were purchased from Invitrogen (Carlsbad, USA) and pustulan from Invivogen Company (San Diego, CA, USA). Laminarin, pullulan, starch, cellulose, and sucrose were obtained from Sigma (St. Louis, USA). Other chemicals utilized in this study were prepared from Merck (Darmstadt, Germany). *Cohnella* sp. A01 was obtained from shrimp pond waste water at Choebdeh (Abadan, Iran; accession No. JN208862.1)[21]. VMD 1.9 (University of Illinois, Urbana-Champaign, USA), Gene Runner 4.0 (Lynnon Biosoft, Canada), and Graphpad Prism 6 (San Diego, CA, USA) software were used to analyze protein structure and DNA sequence as well as to draw Michaelis-Menten curve and to calculate K_m_ and V_max_. The phylogenetic tree was established using the maximum likelihood method implemented in the PhyML program (v3.1/3.0 aLRT)[22].

### Cloning of Cohnella sp. A01 bg16M gene

*Cohnella* sp. A01 was employed as the gene source. Bacterial cells were cultured in Nutrient broth medium at 60 °C for 3 days. The target gene was amplified by PCR using forward primer with the *Nde*I site (5’-CTTCATATGATGCTTTTGAAGGCGAAATCGAAGTCC-3’; T_m_ = 66.6 °C) and reverse primer with restriction site of *Not*I (5’-CTTGCGGCCGCT TTTCTCACCAGTTCGAAATCGTCGATAC-3’; T_m_ = 68 °C). Ligation reaction between the 1917-bp amplified fragment and the pTZ57R/T vector was performed, and the resulting vector was confirmed by sequencing using the M13/pUC forward and reverse primers. The recombinant plasmid was digested by *Nde*I and *Not*I, and the gene fragment was subcloned into pET 26b (+). *E. coli* BL21 cells were transformed with the final expression construct.

### Expression and purification

After transformation, one positive colony was selected and incubated at 37 °C for 16 h. A volume of 3 ml grown bacteria was added to 50 ml fresh medium containing kanamycin (30 µg/ml), and the culture was incubated at 37 °C until OD at 600 nm reached 0.5. Thereafter, the bacteria were induced at final concentration of 1 mM of isopropyl β-D-1-thiogalactopyranoside and incubated at 30 °C for 6 h. Bacterial cells were then collected by centrifugation (9,000 ×g at 4 °C for 10 min) and were mixed in the binding buffer (50 mM NaH_2_PO_4_, 500 mM NaCl, 0.05% Tween 20, and 10 mM imidazole 50 mM) at pH 8. The resulting solution was incubated at 4 °C for 1 h. Cell walls were lysed using a sonicator (50 MHz and 60% power) at seven cycles, each consisting of a 30-s pulse and 1-min sonication break on ice. The obtained crude cell extract was immediately centrifuged at 16,000 ×g at 4°C for 10 min, and the supernatant was utilized for purification process. Due to the *polyhistidine*-tag at the C-terminal of the recombinant protein, Ni-NTA chromatography method was used for enzyme purification as follows: The obtained supernatant from lysed bacterial cells was passed through the affinity column three times, and the flow-through was collected. Subsequently, the host proteins were added to the column by injecting 5 ml washing buffer (50 mM NaH_2_PO_4_, 500 mM NaCl, 0.05% Tween 20, and 30 mM imidazole 50 mM) at pH 7, and the eluted fractions were collected in the other tubes. At this step, proteins lacking *polyhistidine*-tag were eluted. Finally, the solution buffer (50 mM NaH_2_PO_4_, 500 mM NaCl, 0.05% Tween 20, 100 mM Imidazole 50 mM) was transferred into the column at pH 7, the fractions were collected, and the enzyme activity was measured immediately.

In order to study the enzyme purity, 12% SDS-PAGE prepared by Laemmli[23] method was used. The protein bands were identified by staining with Coomassie brilliant blue R250.

### Enzyme activity and protein concentration assays

The enzyme activity assay was performed by spectrophotometry based on Miller’s[24] method, while pustulan and laminarin were used as substrates. Production of reducing sugar in the reaction solution demonstrated the enzyme activity. The reaction containing 50 μL of 2.5 mg/ml substrate and 50 μL of the purified enzyme was incubated at 50 °C for 30 min. The enzymatic reaction was stopped by the addition of 100 μL of DNS (3,5-dinitrosalicylic acid) and heated at 100 °C for 5 min. The samples were then centrifuged at 7000 ×g at room temperature for 10 min. The reducing sugar concentration was determined by the DNS method, and the absorbance was measured at 540 nm. One unit (U) of bg16M activity is defined as the amount of enzyme that released 1 µmol glucose (as reducing sugar equivalent) per 1 ml per minute at optimum conditions. The measurement of protein concentration was carried out by Bradford method[25] using bovine serum albumin as the standard solution.

### Studying the substrate specification of the bg16M

β-glucans-like laminarin, pustulan, cellulose (β-1,4-glucan) and α-glucans-like starch (α-1,4-1,6-glucan) and pullulan (α-1,4-1,6-glucan) and also disaccharide-like sucrose (O-α-D-glucopyranosyl- (1 → 2)-β-D-fructofuranoside) were applied in order to determine the substrate characteristics of the studied enzyme. Each substrate (2.5 mg/ml) was prepared in 50 µl of the 20 mM reaction buffer at pH 7. Then 50 µl glucanase was added to each substrate and incubated at 50 °C. After 30 min, 100 µl of DNS solution was added. Microtubes containing the reaction solution and DNS were boiled for 5 min, and after cooling, absorbance was measured at 540 nm.

### Effect of pH on stability and activity of bg16M

Laminarin and pustulan were utilized in the 100 mM mixed buffer (acetate-phosphate-glycine) as a substrate with a pH range of 3 to 12 so as to determine pH profile. To investigate the enzyme pH stability, bg16M was placed at a pH range of 3 to 12 for 90 min, and thereafter the enzyme activity was measured under optimum conditions. The enzyme was also placed at pH 3, 8, and 12 for 180 min, and the enzyme activity was measured every 20 min.

### Effect of temperature on stability and activity of bg16M

To determine optimum temperature, the enzyme activity was determined between 30 to 90 °C. Laminarin and pustulan were utilized as substrate in optimum pH. For heat stability studies, the enzyme was incubated at 20-90 °C for 90 min. Thereafter, enzyme assay was carried out in optimum conditions.

### Studying the effect of metal ions and chemical compounds

The effect of metal ions (10 mM), including Na^+^, Zn^2 +^, Cu^2 +^, Fe^2 +^, Mg^2 +^, K^+^, Ca^2 +^, Ni^2 +^, Ba^2 +^, and Ni^2+^, as well as either 10 or 20 mM EDTA, iodoacetate, ammonium sulfate, detergents (Tween 20, Triton X-100, SDS, and urea) on the enzyme activity was studied. For this purpose, the enzyme solution was exposed to these compounds at room temperature for 120 min, and then enzymatic activity was measured.

### Michaelis-Menten constants

Pustulan and laminarin were prepared with the concentrations of 1 to 6 mg/ml, and enzyme activity assay was performed under optimum conditions. For each concentration of substrates, the amounts of released glucose were calculated based on µmol/ml. Graphpad Prism 6 software was applied in order to draw the Michaelis-Menten diagram, and V_max_ and K_m_ were then calculated.

## RESULTS

**Fig. 1:**
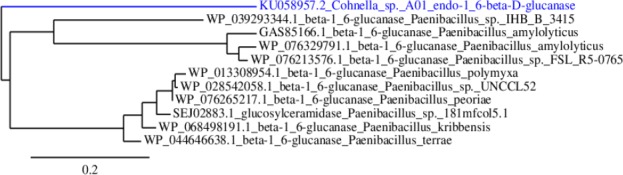
Phylogenetic analysis of bg16M amino acid sequence by similar protein sequences. Maximum likelihood method was implemented in the PhyML software.

**Fig. 2:**
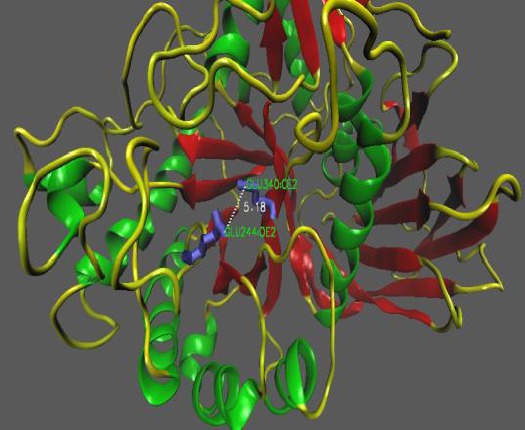
Glutamate (Glu) 244 and glutamate 340 in the enzyme active site, which had an appropriate distance (5.18 Å) in βα 3 and βα 6 loops.

Cloning of the target gene in pTZ57R/T vector was confirmed by sequencing, and in the next step, it was cloned in the pET-26b (+) (MS6911) expression vector successfully (GenBank accession No. KU058957.2). Sequence analysis using the Gene Runner revealed that the gene fragment encodes a protein containing 600 amino acids with a molecular weight of 64.8 kDa. The Protein BLAST was performed to find similar protein sequences. Phylogenetic analysis of bg16M amino acid sequence by similar protein sequences showed a single clade, and no significant similarity to other β-glucanase was found (Fig. 1). Data obtained from amino acid sequence alignment in NCBI database anticipated that the sequence similarity of bg16M corresponded with hydrolase family. Alignment of the remaining fragments also showed that the sequence of 485 to 639 is very similar to the CBM-4-9 family, which includes the proteins with binding site to carbohydrates. Comparing the amino acid sequences of bg16M with those of GH30 proteins revealed five glutamic acid and aspartic acid residue, which are conserved in all β-glucanase in GH30 family such that glutamate 340 and glutamate 244 from the active site had an appropriate distance (Fig. 2). The Phyre2 (http://www.sbg.bio.ic.ac.uk/phyre2) was used to predict 3D structure. The resulting structure was determined using VMD software (ver. 1.9.2).

### Purification of recombinant enzyme

**Fig. 3:**
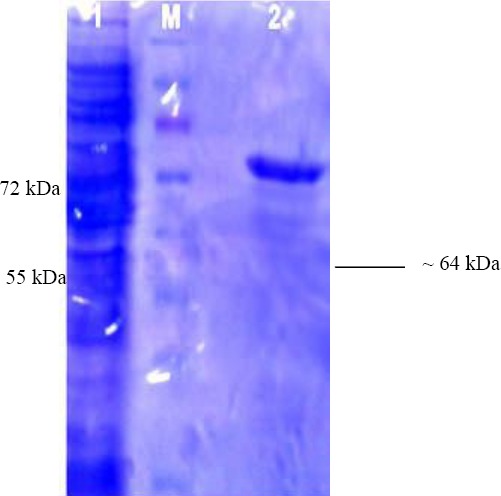
β-1,6-glucanase SDS-PAGE. Lane 1, the bacteria were induced at the final concentration of 1 mM of IPTG and incubated at 30 °C for 6 h; lane M, protein marker; lane 2, purified enzyme.

The enzyme was purified utilizing the Ni-NTA column and dialyzed against phosphate-buffer saline at pH 7.5 for 20 h. The purity of the enzyme was analyzed by SDS-PAGE (Fig. 3). The recombinant protein was purified with a yield of almost 48% (Table 1). The molecular weight of bg16M was found to be around 65 kDa.

**Table 1:** The efficiency of purification of recombinant β-1,6 glucanase

Steps	Activity (unit)		Specific activity (U/mg)		Protein (mg/ml)	Purification (fold)	Yield (%)
	
	Laminarin	Pustulan	Laminarin	Pustulan			
Crude	4.83	2.88	4.92	2.93	0.98	1	100
Ni-NTA srpharose	2.24	1.41	7	4.4	0.32	1.5	46.37

### Enzyme substrate specification

**Fig. 4:**
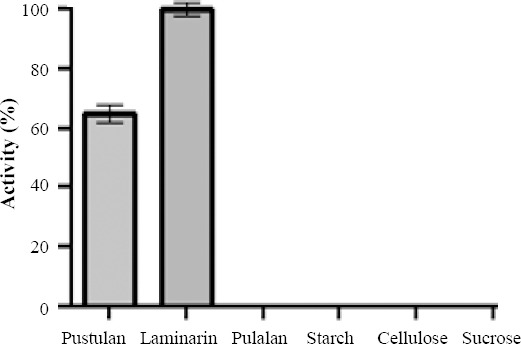
Substrate specificity of β-1,6-glucanase. The bg16M activity on laminarin was 100%. It also showed a relative activity on pustulan, but no activity on other substrates.

Glucanase substrate specification in the presence of β-glucan-like laminarin, pustulan, cellulose, starch, and pullulan, as well as the disaccharides such as sucrose was measured in triplicates at 50 °C and pH 7. Thereafter, graph of the average activity of the enzyme in the presence of different substrates was drawn by GraphPad Prism (ver. 6) software. bg16M activity on laminarin was found to be 100%, and it showed a relative activity on pustulan, but no activity on other substrates (Fig. 4).

### bg16M pH profile and stability

**Fig. 5:**
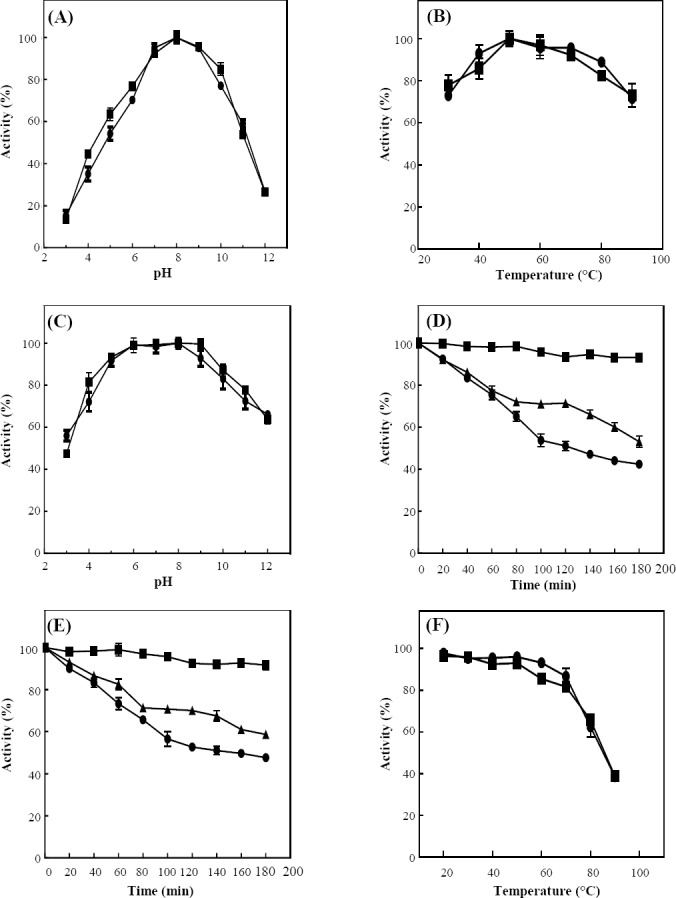
Effect of pH and temperature on activity of bg16M. Laminarin (■) and pustulan (●) were used as substrates. To determine optimum pH (A) and temperature (B), enzymes activities were determined between pHs 3 and 12 and at 30 and 90 °C, respectively. To investigate the stability of the enzyme at different pHs, enzyme was placed at a pH ranges of 3 to 12 for 90 min, and thereafter the enzyme activity was measured under optimum conditions (C). The enzymes were exposed to different pHs, including 3 (●), 8 (■), and 12 (▲) for 180 min, and the enzyme activity was measured every 20 min (D for laminarin and E for pustulan). To study temperature stability, enzyme was incubated at 20 to 90°C for 90 min. Thereafter, enzyme assay was carried out in optimum conditions (F).

The results of pH on the enzyme activity and stability revealed that the recombinant bg16M had the highest activity at pH 8, and the pH ranges of 7 to 9 were the desirable pH for the activity of this enzyme (Fig. 5A). The highest stability of the bg16M was also observed at pHs 8 and 9. The remaining activity of enzyme, after 180 min of incubation at pHs 3, 8, 12 was 42, 93, and 53%, respectively, indicating that this enzyme is stable in a wide range of pH (Fig. 5C and 5D).

### bg16M temperature profile and stability

The bg16M showed the best activity at 50 °C. This enzyme revealed over 80% activity at temperatures between 40 and 70 °C (Fig. 5B). The bg16M also indicated more than 60% activity at 90 °C. Temperature stability at 20 to 90 °C was carried out for 90 min. The results demonstrated that bg16M activity at 20, 30, 40, and 50 °C were above 95%, and at 90 °C, 30% of the enzyme activity remained. However, after 120 min, more than 20% of its activity was maintained (Fig. 5E and 5F).

### Studying the effect of cofactors and some chemical compounds

**Fig. 6:**
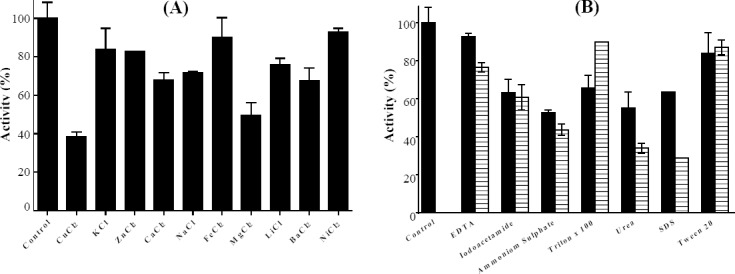
The effect of metal ions and some chemical compounds on the enzyme activity. (A) Metal ions (10 mM) including Na^+^, Zn^2 +^, Cu^2 +^, Fe^2 +^, Mg^2 +^, K^+^, Ca^2 +^, Ni^2 +^, Ba^2 +^, and Ni^2+^, as well as (B) either 10 (ν) or 20 () mM EDTA, iodoacetate, ammonium sulfate, detergents such as Triton X-100, urea, SDS, and Tween 20 was studied.

The results of metal ions effects on residual activity of bg16M indicated that none of the metal ions increases the activity of the enzyme. The highest level of inhibitory effect was originated from magnesium and copper ions, which reduced the enzyme activity by 55 and 64%, respectively. Enzyme maintains more than 60% of its activities in the presence of sodium,

calcium, barium, and lithium ions. However, the lowest inhibitory effect was obtained from nickel, iron, zinc, and potassium, and the remaining activity of the enzyme was more than 80% (Fig. 6A).

The bg16M revealed 90 and 76% of its activity in 5 and 10 mM EDTA, respectively. Hence, since the metal ions had no increasing effects on the enzyme activity and also due to the enzyme activity in the presence of EDTA, it can be concluded that this is not a metalloenzyme. Iodoacetamide blocks cysteine residue in the activation site of the enzyme. This compound causes a 40% reduction in the enzymatic activity of bg16M. The 5 and 10 mM concentrations of ammonium sulfate led to 50 and 40% reductions in the remaining activity of bg16M, which is probably due to low solubility of the enzyme. Also, 5 and 10 mM SDS reduced the remaining bg16M activity by 35 and 75%, while reduction of enzyme activity after treatment with urea was 45 and 65%, indicating that 10 mM SDS has more effect on enzyme activity. Reduction of enzyme activity by Tween 20 in both concentrations of 5 and 10 mM was less than 20%, while Triton X-100 reduced bg16M activity by 35 and 10%. To the best of our knowledge, studying the effect Triton X-100 and Tween 20 on the glucanase enzymes is yet to be discovered (Fig. 5B).

### Determination of kinetic parameters

**Fig. 7:**
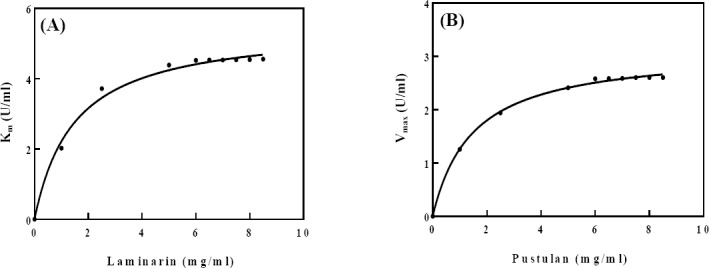
Michaelis-Menten curve of bg16M activity versus different concentrations of laminarin (A) and pustulan (B) incubated at the optimum conditions. The K_m_ and V_max_ values of the enzyme for laminarin and pustulan were 1.2 mg/ml and 4.83 U/ml (A) and 1.08 mg/ml and 2.88 U/ml (B), respectively.

The K_m_ of the enzyme for pustulan and laminarin was 1.08 and 1.20 mg/ml, but V_max_ of the enzyme for pustulan and laminarin were 2.88 and 4.83 U/ml, respectively (Fig. 7).

## DISCUSSION

In the present research, *Cohnella sp*. A01 *bg16M* gene was cloned in an expression vector and was expressed in *E. coli*, and then its purification and characterization were performed. The molecular weight of bg16M was obtained as approximately 65 kDa. The bg16M in *Trichoderma harzianum* and *Streptomyces* sp. EF-14 and *Penicillium multicolor* had molecular weights about 43 and 51 kDa, respectively[13,26,27].

Laminarin is the best substrate for bg16M activity, and it had a relative activity on pustulan. Other enzymes had activity have a ctivity on both substrates have also been reported[26]. *T. harzianum* bg16M enzyme had no effects on the glucans extracted from *S*. *cerevisiae*, which mainly composed of β-1,3-1,6-glucose units, while its activity on pustulan and laminarin was 31 and 86 U/mg, respectively. It further demonstrated almost no activity on other substrates[13]. *Streptomyces* sp. The bg16M had no activity on laminarin, which may be due to inability of the enzyme to recognize β-1,6 bond near the 1, 3-glycosidic bonds. In other words, these enzymes require the substrates that are all β-1,6-glycosidic bonds with no gap between[26]. Table 2 shows heterogenesity of some bg16M enzymes. bg16M had the highest activity at pH 8. The remarkable point is that the most studied bg16M have had the optimal activity at pHs between 4 and 5.6. For instance, bg16M extracted from the *P. brefeldianum* had optimum activities at pH 2.4[28], bg16M from *Bacillus circulans* WL-12 showed the best activity at pH 5.6-6[29], bg16M from *Acinetobacter*, and *Streptomyces* sp. had optimum activity at pH 5.5. Enzyme from *P. multicolor* showed optimal activity at pH 4; nevertheless, no bg16M was observed with the optimal activity in the alkaline conditions[26,27,30].

**Table 2:** Some β-glucanase heterogenesity

Enzyme sources	Ability to hydrolyze laminarin	Ability to hydrolyze pustulan	Molecular weight (kDa)	Ref.
*Trichaderma harzianum* (BGN 16.1 glucanase)	Yes	No	51.0	[42]
*Trichaderma harzianum* (BGN 16.2 glucanase)	No	Yes	43.0	[13]
*Acremonium persicinum*	Yes	Yes	42.7	[31]
*Bacillus circulans* WL-12	No	Yes	52.0	[16]
*Streptomyces* sp. EF-14	No	Yes	65.0	[26]
*Penicillium multicolor*	No	Yes	51.0	[27]
*Acremonium sp.* IMI 383068	Yes	Yes	41.2	[33]
*Cohnell sp. A01 β glucanase*	Yes	Yes	65.0	This study

Stability and optimal activity at alkaline pH are an appropriate feature for function of the enzyme on harmful microorganisms (β-1,6-glucan is a cell wall) in the human intestine, which has an alkaline pH. Evaluation of the pH stability of other bg16M revealed that the majority of enzymes are mostly stable in the acidic pHs of 3 to 6, with few exceptions, such as bg16M enzyme in *A. persicinum* which has stability in the range of 5.4-5.9[31] *N. crassa*[32] and *P. brefeldianum* that are stable in the pH ranges of 5 to 8, and bg16M from *P. multicolor* shows an appropriate stability in the pH ranges of 2 to 9[28].

Our experiments revealed that 50 °C is the bg16M optimum temperature, and the enzyme maintained over 80% of activity up to 70 °C. The optimum temperature for the activity of *B. circulans* WL-12 bg16M was 45 °C, for *T. harzianum* bg16M was 50 °C[13], and for other studied bg16Ms were between 40 and 60 °C[29]. The optimum temperature of bg16M purified from the *P. brefeldianum* was 50 °C, though enzyme activity was reduced to less than 10% at 80 °C[25]. The optimum temperature of the enzyme extracted from *Streptomyces* sp. has been reported to be at 55 °C[26], in *P. multicolor* is 50 °C[27], and in *Acremonium* sp., the optimal activity of IMI 383068 was observed at 40 °C in pH 5 and at 50 °C in pH 5.6[33].

Remaining enzyme stability after its incubation in different temperatures was remarkable. Interestingly, bg16M purified from the *P. brefeldianum* showed low stability at higher than 37 °C, while it lost enzymatic activity after 60 min at 60 °C[28]. The *Streptomyces* sp. bg16M was unstable at temperatures higher than 45 °C[26], *P. multicolor* enzyme was unstable at above 60 °C[27], and *Acremonium* sp. IMI 383068 enzyme was also unstable in over 75 °C[33].

β-glucanases belonging to GH30 enzyme family do not need a cofactor to carry out their normal functions, and heavy metal ions act as inhibitors or activity-reducing agents for this family. The effect of 1 mM magnesium, copper, and zinc on the bg16M from the filamentous fungus *A. persicinum* revealed the remaining enzyme activity of 86, 20, and 44%. Furthermore, the metal ions including barium, lithium, sodium, potassium, iron, and nickel had a slightly effect on enzyme activity at the concentrations of 1 to 20 mM[31]. The inhibitory effect of copper on the bg16M has been approved in several other microorganisms with different rates[34,35]. According to the available reports, 5 mM EDTA reduces the activity of metalloenzymes by chelating the metal ions[36,37]. The cause of a slight reduction in the enzyme activity is likely due to the effect of EDTA on the structure of enzyme or active site.

Ammonium sulfate is commonly utilized to precipitate proteins by protecting their normal structures and long-term maintenance of many proteins. Protein solubility is because of its ionic interaction with the solvent and since ammonium sulfate reduces these interactions, without irreversible denaturing, it leads to partial precipitation of protein. There is no available report on the inhibitory effect of ammonium sulfate on bg16M activities[38].

Detergent-like SDS and urea are widely used for protein denaturation; however, resistance of proteins to these detergents is different. Despite the wide use of urea as a denaturant, the molecular mechanism of its denaturation activity is not clear yet. Urea may directly bind to protein or indirectly change the environment of reaction solution or further prevent protein re-folding and fix the denatured status[39]. Thermophilic proteins are more stable than their mesophyll counterparts in urea effects. Triton X-100 and Tween 20 are non-ionic surfactants having a hydrophilic polyethylene tail and lipophilic aromatic heads used as detergents. The most important application of these surfactants is their interaction for re-folding the proteins and removing the inclusion bodies of proteins[40].

Kinetic study of bg16M revealed that this enzyme is consistent with the Michaelis-Menten model[2,6]. The bg16M indicates greater affinity for pustulan. Higher V_max_ value for laminarin is an exception, due to the fact that the β-1,6-glycosidic bonds of laminarin is almost a quarter of its total β-glycoside bonds. Nevertheless, pustulan has fully formed the β-1,6-glycosidic bonds. The reason for this difference is probably due to better replacement of laminarin in the enzyme active site or due to small size and high solubility of laminarin, when compared to pustulan. The kinetic parameters of bg16M from different organisms for pustulan are: *B. circulans* WL-12 V_max_ = 8.8 U/mg and K_m_ = 29.0 mg/ml; *T. harzianum* V_max_ = 224 U/mg and K_m_ = 4.2 mg/ml; *Streptomyces sp*. V_max_ = 284 U/mg and K_m_ = 19.0 mg/ml[26,29].

An advantage of industrial application of enzymes is environmental considerations as opposed to chemical catalysts[41]. Biochemical characterization of this novel bg16M suggests that this enzyme can be applied in beverage industries and medical sectors because of its high activity and thermal stability, as well as its stability and ability to act in the presence of alkali pH, metal ions, and most detergents.
